# *Ferula ammoniacum* gum aqueous extract exerts anti-inflammatory and antioxidant mechanisms to combat aluminum chloride-induced Alzheimer’s disease in rats: possible involvement of opioid pathways

**DOI:** 10.3389/fphar.2025.1708643

**Published:** 2026-01-02

**Authors:** Sajad Fakhri, Mohammad Mehdi Gravandi, Mohammad Bagher Majnooni, Mohammad Hosein Farzaei, Fatemeh Abbaszadeh, Khodabakhsh Rashidi, Javier Echeverría

**Affiliations:** 1 Pharmaceutical Sciences Research Center, Kermanshah University of Medical Sciences, Kermanshah, Iran; 2 Student Research Committee, Kermanshah University of Medical Sciences, Kermanshah, Iran; 3 Neurobiology Research Center, Institute of Neuroscience and Cognition, Shahid Beheshti University of Medical Sciences, Tehran, Iran; 4 Research Center of Oils and Fats, Kermanshah University of Medical Sciences, Kermanshah, Iran; 5 Departamento de Ciencias del Ambiente, Facultad de Química y Biología, Universidad de Santiago de Chile, Santiago, Chile

**Keywords:** *Ferula ammoniacum* gum, aqueous extract, neuroprotection, Alzheimer’s disease, aluminum chloride, oxidative stress, opioidergic pathway

## Abstract

**Background:**

Alzheimer’s disease (AD), the most common form of dementia, significantly affects memory and behavior due to dysregulated pathways involving oxidative stress, inflammation, and opioidergic systems. Currently, no effective treatments are available, underscoring the need for novel alternatives. *Ferula ammoniacum* (D.Don) Spalik, M. Panahi, Piwczyński, and Puchałka [Apiaceae] (FA), an Iranian medicinal plant, is known for its anti-seizure, anti-inflammatory, and analgesic properties, with its gum utilized as a nerve tonic.

**Purpose:**

This study investigates the anti-AD effects of *F. ammoniacum* gum aqueous extract (FAGAE) using an aluminum chloride (AlCl_3_)-induced Wistar rat model of AD.

**Materials and methods:**

The aqueous extract, prepared by macerating powdered gum in distilled water for 48 h at ambient temperature, was subjected to phytochemical analysis using ultraviolet, infrared, and nuclear magnetic resonance spectroscopy. Thirty rats were assigned to five different groups: one receiving saline, one receiving AlCl_3_ (100 mg/kg, i.p.), two receiving AlCl_3_ followed by oral treatment with FAGAE at doses of 50 or 100 mg/kg, and one receiving naloxone (an opioid receptor antagonist) along with AlCl_3_ and the effective dose of FAGAE. Behavioral changes were evaluated using the open-field, passive avoidance, and elevated plus maze tests. Furthermore, biochemical analyses were conducted to measure the serum nitrite levels, changes in weight, matrix metalloproteinase (MMP) activity, and histopathological changes in brain tissue.

**Results and discussion:**

The phytochemical analysis of FAGAE revealed the presence of polysaccharide compounds with tentative arabinogalactan structures. FAGAE decreased step-through latency in the passive avoidance test and modified AlCl_3_-induced weight changes. FAGAE also significantly increased mobility, grooming, and crossing in the open-field test. Naloxone reversed the anti-AD effects of FAGAE, suggesting a possible role for opioidergic pathways in its therapeutic effects. Zymography results showed that FAGAE reduced MMP-9 activity while increasing MMP-2 activity. Histopathological analysis revealed a preserved number of intact neurons in the hippocampus, whereas reduced serum nitrite levels were observed after FAGAE administration in rats with AD.

**Conclusion:**

Behavioral, biochemical, and histopathological impairments induced by AlCl_3_ were significantly attenuated by FAGAE, possibly through the opioidergic pathway, which combats inflammation and oxidative stress and supports neuronal survival.

## 1 Introduction

Alzheimer’s disease (AD) is a neurodegenerative disorder characterized by memory and cognitive deficits and histopathological and neurobehavioral changes. It is distinguished by unchecked development and accumulation of the pathogenic amyloid-beta (Aβ) peptide, which is produced as a result of the consecutive activity of beta- and gamma-secretases on the amyloid precursor protein (APP) (Cam and Bu, 2006). Maintaining metal particle homeostasis in the brain is essential for normal cognitive function; however, dysregulation of this homeostasis has been a pivotal step in the progression of neurodegeneration. Such dysregulated events are interconnected with oxidative stress and opioidergic pathways (Iranpanah et al., 2024). As critical regulators, opioid receptors play modulatory roles in brain tissue in the recovery of functional and memory impairments, cognitive decline, and local cerebral ischemia in animal models (Chen et al., 2016). Opioid receptors have also been targeted to regulate cognitive functions (Song et al., 2021).

External administration of high-dose aluminum chloride (AlCl_3_) is known to accelerate the oxidative stress and generation/aggregation of extracellular Aβ (Drago et al., 2007). Additionally, AlCl_3_ alters cholinergic activity and oxidative stress levels, which are key events in the neurochemistry of AD (Rakonczay et al., 2005). As a critical hypothesis in AD, destruction of cholinergic pathways in the basal forebrain and cerebral cortex leads to associated signs and symptoms. Accordingly, AlCl_3_ triggers cascades of cholinergic pathways, oxidative stress, and inflammatory pathways (Hampel et al., 2019). The lower efficacy of conventional drugs (e.g., donepezil, rivastigmine, physostigmine, and memantine) against AD, along with their considerable side effects, limits associated applications (Schliebs, 2005; Abbas Raja et al., 2025). Consequently, a global effort has been made to use herbal medicines, which are more effective and have fewer side effects. Additionally, epidemiological studies have shown a link between the consumption of multi-targeting plant-derived therapeutic agents and several health benefits (Espín et al., 2007).

*Ferula ammoniacum* (D.Don) Spalik, M. Panahi, Piwczyński, and Puchałka (syn. *Dorema ammoniacum*) [Apiaceae] (FA) is a perennial plant containing a multitude of chemical constituents and has traditionally been used to treat various diseases (Motevalian et al., 2017). Additionally, gum ammoniacum, a naturally oozing oleo-gum-resin latex, is found in the cavities, stems, roots, and petioles of this plant. The expectorant, antispasmodic, diaphoretic, carminative, moderate diuretic, poultice, antibacterial, spleen and liver tonic, anticancer, and vasodilator properties of FA are attributed to its resin (Sharafzadeh and Alizadeh, 2012; Javadi et al., 2015; Naghibi et al., 2015). Gum ammoniacum is still used in Western and Indian medicine for the treatment of chronic bronchitis and persistent coughs. It is recognized in the British Pharmacopoeia as an antispasmodic and expectorant herb (Hosseini et al., 2014). FA contains several phytochemicals, including terpenes, coumarins, and various other phenolic compounds. Active constituents of FA exhibit multiple therapeutic activities, including anti-inflammatory, antimicrobial, and antioxidant effects (Zibaee et al., 2020). Some isolated FA compounds have also shown acetylcholinesterase inhibitory activity (Adhami et al., 2013; Nazir et al., 2021). From a mechanistic perspective, FA plays a critical role in improving antioxidant capacity, serving as a line of defense against the oxidative pathway and balancing oxidative mediators (Delnavazi et al., 2014). Therefore, considering the involvement of oxidative stress, inflammation, and opioidergic pathways in the pathogenesis of AD, and the multi-targeting potential of FA, it could be a promising agent in combating AD (Abraki and Chavoshi-Nezhad, 2014; Ghorbani et al., 2020; Speer et al., 2020; Al-Obaidi et al., 2021). The present study was carried out to explore the neuroprotective effect of FA gum aqueous extract (FAGAE) against AlCl_3_-induced AD in rats, focusing on its anti-inflammatory and antioxidative effects, which appear to act through opioid receptors.

## 2 Materials and Methods

### 2.1 Plant material

*Ferula ammoniacum* (D.Don) Spalik, M. Panahi, Piwczyński, and Puchałka [Apiaceae] was harvested in the Kermanshah Province, Iran, between July and August 2020. Herbarium specialists from Razi University in Kermanshah, Iran, Division of Botany, Department of Biology, School of Science, further validated the dry gum.

### 2.2 Preparation of the aqueous extract

The *F. ammoniacum* gum (FAG) was cleaned, cut, and dried in air before being powdered. Approximately 100 g of the dry powder was macerated in 500 mL of water for 48 h with continuous stirring, after which it was filtered through muslin cloth, followed by paper filtration. A freeze dryer was then used to dry the extract used for chemical characterization according to ConPhyMP (Heinrich et al., 2022).

### 2.3 Structural characterization by UV, FT-IR, and NMR spectroscopy

An amount of 2 mg of freeze-dried FAGAE powder was mixed with 100 mg of KBr (FT-IR grade) and compressed to form a 3-mm-diameter salt disc. This disc was loaded onto an FT-IR spectrometer (IR Prestige-21, Shimadzu, Japan), and spectra were scanned over 400 cm^-1^–4,000 cm^-1^ at a resolution of 4 cm^-1^. Additionally, FAGAE was subjected to UV–VIS (CPS-240A, Shimadzu, Japan) scanning analysis. The absorbance spectra were recorded between 200 and 900 nm using a UV–VIS spectrophotometer (Keskin et al., 2024). ^1^H and ^13^C nuclear magnetic resonance (NMR) spectra of the FAGAE solutions in D_2_O (Mesbah Energy, Iran) were recorded at ambient temperature using a 400 MHz Avance Bruker spectrometer (Bruker BioSpin, Rheinstetten, Germany). ^1^H NMR was operated at 400 MHz, and ^13^C NMR was operated at 100 MHz.

### 2.4 Animals

Healthy male Wistar rats (220 g–250 g) were procured from the central animal house at Kermanshah University of Medical Sciences and maintained under a 12/12 h light/dark cycle, 60% ± 5% relative humidity, and 24 °C ± 2 °C temperature, with food and water *ad libitum*. Additionally, all experimental procedures were carried out in accordance with the institution’s approved guidelines and the animal care and ethics principles of Kermanshah College of Medical Sciences, Iran (Ethical code: IR.KUMS.REC.1399.481). Altogether, thirty rats were randomly divided into five groups of six for 14 days (each group of three rats was housed in a separate cage). Group I received intraperitoneal (i.p.) saline and oral distilled water. Group II received AlCl_3_ (100 mg/kg, i.p.) and distilled water orally (negative control). Groups III and IV received AlCl_3_ (100 mg/kg, i.p.), followed by oral administration of FAGAE (50 mg/kg) and FAGAE (100 mg/kg). Group V received AlCl_3_ (100 mg/kg, i.p.), followed by naloxone (0.1 mg/kg, i.p.) + oral administration of FAGAE (50 mg/kg). All agents were administered i.p. and daily. AlCl_3_ was provided by Sigma-Aldrich (St. Louis, Missouri, United States).

Based on several animal reports on AD and considering the multiple dysregulated mechanisms involved, along with the limited efficacy of current interventions, the inclusion of a positive control was not necessary. Additionally, no data were excluded during the study, and all the obtained data were used for analysis.

### 2.5 Experimental design

At the end of the experimental period, animals were analyzed for their memory, movement, and motor activity in the open-field, passive avoidance, and elevated plus maze tests. Rats were then euthanized with ketamine (100 mg/kg) and xylazine (20 mg/kg), and serum was used for nitrite measurement and zymography. Additionally, the hippocampus was excised for histopathological analysis. All the experiments were performed in a blinded manner. Blood samples were collected from the retro-orbital sinus of the rats (Zamani et al., 2025).

### 2.6 Behavioral studies

#### 2.6.1 Open-field test

The open-field test is one of the most common methods for quantitatively and qualitatively measuring exploratory behavior and automatic motor behavior (Peng et al., 2021). The floor of the wooden apparatus (W 100 cm x D 100 cm x H 40 cm) was enveloped with resin and separated into 25 squares (5 x 5). The rats were fitted in one corner of the open-field chamber, and the behavior changes were detected for 5 minutes to measure the following parameters: (a) numbers of grooming, which included licking, washing face, or scratching behavior on days 13 and 14; (b) numbers of squares explored, which included entering the peripheral or central squares with both forelimbs on days 13 and 14; and (c) numbers of rearing, which included vertical exploration on days 13 and 14 (Biedermann et al., 2017; Kraeuter et al., 2019). Then, the differences between the results of days 13 and 14 were reported.

#### 2.6.2 Passive avoidance test

The passive avoidance test is one of the behavioral tests in which the animal learns to avoid electrical stimuli (Nassiri-Asl et al., 2010; Sohn et al., 2020). The passive avoidance test, commonly used to assess long-term memory, was carried out according to the protocol described by Yamaguchi and Kawashima (2001). The device had two identical compartments, separated by a guillotine door. Two chambers had light and dark opaque resin for the walls and floors. Each rat was placed on the device on the first day of testing and kept there for 5 minutes, allowing it to become accustomed to freely exploring it without being shocked by electricity. The rats were put in the light chamber to conduct the acquisition and retention trial. After 60 s of habituation, the guillotine entryway was opened, and the initial latency (IL), to enter the dull chamber, was recorded. The door was shut as soon as the rat entered the dimly lit space, and an isolated stimulator delivered an unavoidable scrambled single-foot electric shock (50 Hz, 0.2 mA, 3 s; once) through the grid floor. Rats with an IL more prominent than 60 s were excluded. On the test day, the interval between the situation within the light chamber and the passage into the dark chamber was measured as strep-through latency (STL) with a cut-off time of 300 s (Iranpanah et al., 2024). Although both IL (on the training day) and STL (on the test day) measure the time it takes for rats to enter the dark compartment, they reflect different cognitive processes. While IL presents the baseline exploration + learning ability on the training day, STL shows memory retention of the aversive experience, which is usually inverse (Rastinpour et al., 2025; Zarneshan et al., 2025).

#### 2.6.3 Elevated plus maze test

The elevated plus maze test is used to assess spatial learning and memory and is primarily used to quantify anxiety in rodents (Itoh et al., 1990; Bernardina et al., 2021; Morikawa et al., 2021). The elevated plus maze consisted of two opposed open arms, separated by two identical closed walls (40 cm high). The central square united the open arms, while the maze itself was suspended 50 cm above the ground. Rats were individually positioned at one end of the open arm on day 14. The initial transfer latency (ITL) was measured as the amount of time it took the animal to transition from the open arm to the closed arm in the maze.

### 2.7 Body weight changes

The rats were weighed on day 14 at the beginning of the experiments. The changes in the rat’s weight were computed in each group using the formula below:

Weight difference = (animal’s weight on day 14 − weight on day 0).

### 2.8 Nitrite assay

Because nitric oxide (NO) is unstable, it is measured as stable metabolites—nitrate and nitrite—using the Griess reaction (Sullivan et al., 1977). The deproteinization step is essential for serum and plasma samples in the Griess reaction. Using the zinc sulfate sedimentation method is the best approach compared to other methods. In the Griess reaction, nitrite combines with sulfanilamide (2 g in 100 mL of 5% hydrochloric acid) as an unstable salt and a mediator, allowing nitrite to react with another agent, such as *N*-naphthyl-ethylenediamine dihydrochloride (NEED, 0.1 g in 100 mL of distilled water). This reaction produces a purple spectrum that can be used to measure nitrite concentration.

For deproteinization, 6 mg of zinc sulfate powder was mixed with 400 µL of the serum samples, and the mixture was vortexed for 1 min. After mixing, the samples were centrifuged at 1,000 *g* for 10 min at 4 °C, and the supernatant was collected. Then, 100 µL of sodium nitrite standards were added to the first eight wells. In the second row, 100 µL of serum was added. Then, 50 µL of sulfanilamide solution was added, and after 5 minutes, NEED was added to the serum and standards. The microplate was incubated at 37 °C for 30 min. Subsequently, the range of purple colors produced by absorption was measure using an ELISA reader with 540 nm and 630 nm reference filters. In the final step, the standard curve was drawn to measure the nitrite level.

### 2.9 Zymography

Zymography was used to measure matrix metalloproteinase (MMP) activity. Serum with a total protein concentration of 100 μg was loaded onto sodium dodecyl sulfate–polyacrylamide gels containing 0.1% gelatin. Electrophoresis was performed at 150 V, followed by a renaturation step in a buffer containing 2.5% Triton X-100 in 50 mM Tris-HCl. The gel was then incubated at 37 °C in a buffer with NaN_3_, NaCl, and CaCl_2_ in Tris-HCl for 18 h. Following incubation, the gel was stained with Coomassie blue and destained in a solution of 5% acetic acid and 7% methanol. The appearance of clear bands on the gel indicated gelatinolytic activity, which was quantified using ImageJ software from the National Institutes of Health (United States of America) (Ren et al., 2017).

### 2.10 Histopathological analysis

Histopathological studies were performed on the hippocampal tissue. Hematoxylin and eosin (H&E) staining was used to evaluate histopathological alterations, which were examined and recorded at ×400 magnification under a light microscope (Inohana et al., 2018). The quantification of neurons in the Cornu Ammonis subregion 1 (CA1), Cornu Ammonis subregion 3 (CA3), and dentate gyrus (DG) regions of the hippocampus was performed using ImageJ software.

### 2.11 Statistical analysis

In the present report, data were analyzed using GraphPad Prism software version 6.0, with one-way analysis of variance (ANOVA), followed by Tukey’s test. The *p-*values <0.05 were considered statistically significant.

## 3 Results

### 3.1 FT-IR and UV–VIS spectroscopy analysis

**FIGURE 1:**
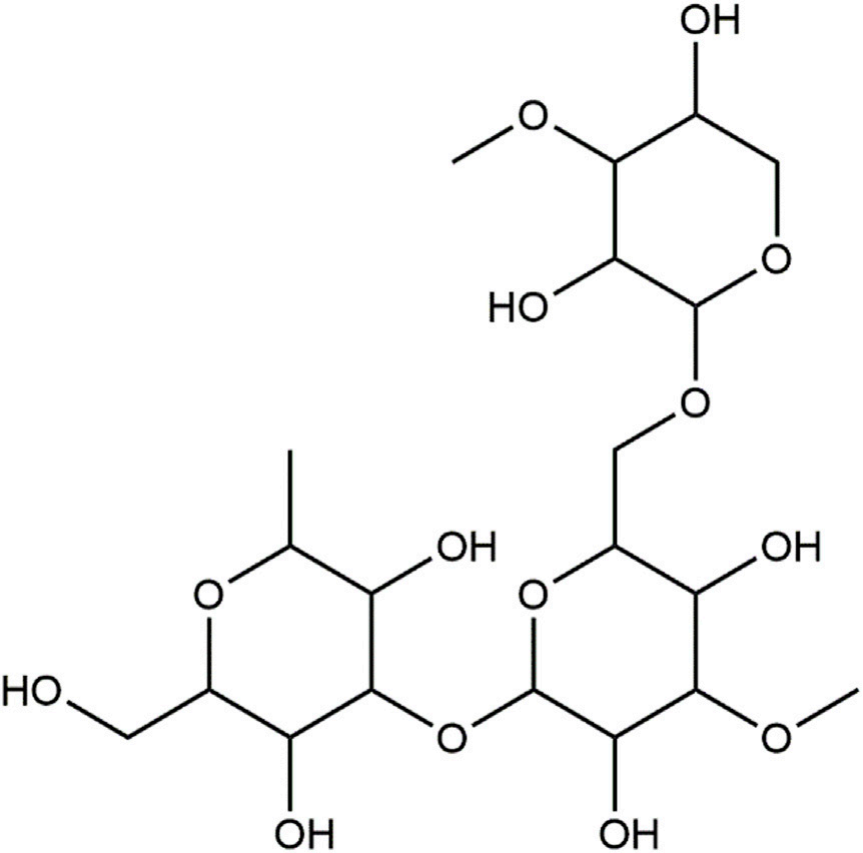
Basic chemical structure of arabinogalactans.

FT-IR analyses of the freeze-dried powder of FAGAE revealed prominent peaks at the range of 3,408 cm^-1^–1,043 cm^-1^, which, according to the other FAGAE phytochemical studies and FT-IR spectra pattern, can be related to the presence of sugars such as oligosaccharides and polysaccharides such as arabinogalactans in this extract (Figure 1) (Ahmadi et al., 2022; Ebrahimi et al., 2022; Ibieta et al., 2023; Saad et al., 2024).

**FIGURE 2:**
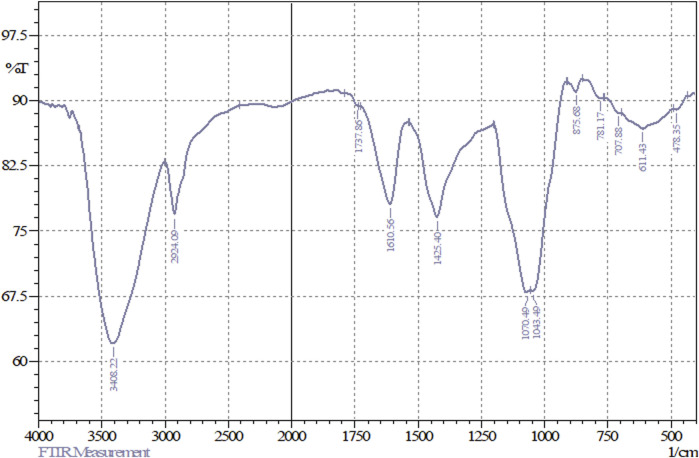
FT-IR spectrum of FAGAE.

The bands at 3,408.22 cm^−1^ and 2,924.09 cm^−1^, respectively, corresponded to the stretching vibrations of O–H and C–H bonds, which form the main skeleton of the chemical structure of sugars. In addition, the bands at 1,070.49 cm^−1^ and 1,043.49 cm^−1^ correspond to the vibrations of C–O and C–O–C groups present in the structure of sugar compounds (Figure 2). The region below 900 cm^−1^ (from 875 cm^−1^ to approximately 700 cm^−1^) represents vibrations from the anomeric region of sugars. The weak bands at 1,737.86 cm^−1^ are attributed to carbonyl (C=O) or carboxyl (CO_2_H) functional groups in sugars such as glucose, fructose, and glucuronic acid. Moreover, the band at 1,610 cm^−1^ could correspond to the water bound to the sugar chains (Kacuráková and Mathlouthi, 1996; Hong et al., 2021).

**FIGURE 3:**
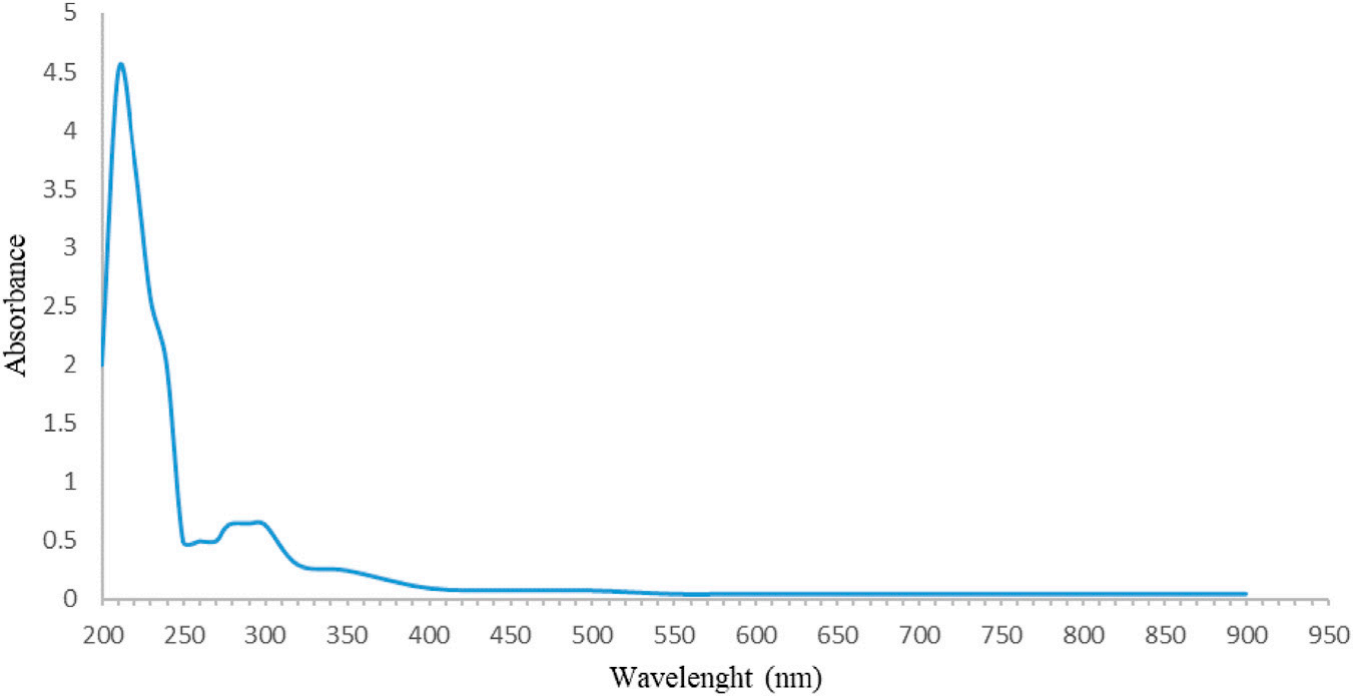
UV–visible spectrum of FAGAE.

The UV spectrophotometry results confirmed the findings of the FT-IR analysis. The maximum absorption in the range of 200 nm–240 nm (Figure 3) and the absence of a prominent UV spectrum at other wavelengths are characteristic of sugars. This maximum absorption can be related to the presence of some functional groups, such as carbonyl (C=O) groups, which are found in sugars (Dias et al., 2009; Trabelsi et al., 2009). According to the studies, sugars were extracted and identified from the main structure of FAG. DAC-1 was isolated and identified as a new polysaccharide with an arabinogalactan structure from FAGAE by Ahmadi et al. (2022). In addition, another novel polysaccharide, 4-*O*-methyl-α-D-glucopyranosyl-branching, was purified from FAGAE by Ebrahimi et al. (2022).

### 3.2 NMR spectroscopy analysis of FAGAE

**FIGURE 4:**
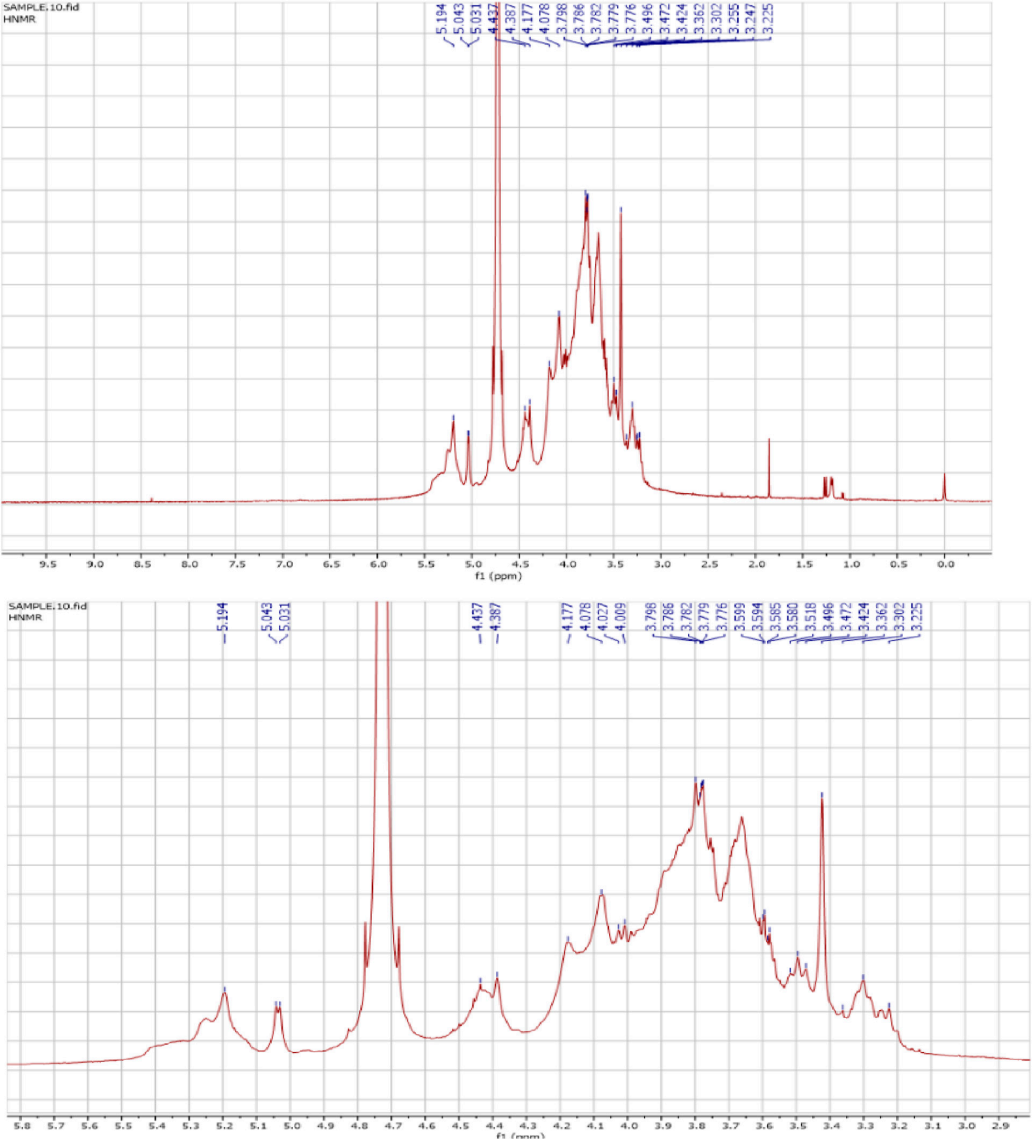
^1^H NMR spectrum of FAGAE. The signal at δ 4.8 ppm corresponds to HDO in the solvent.

**FIGURE 5:**
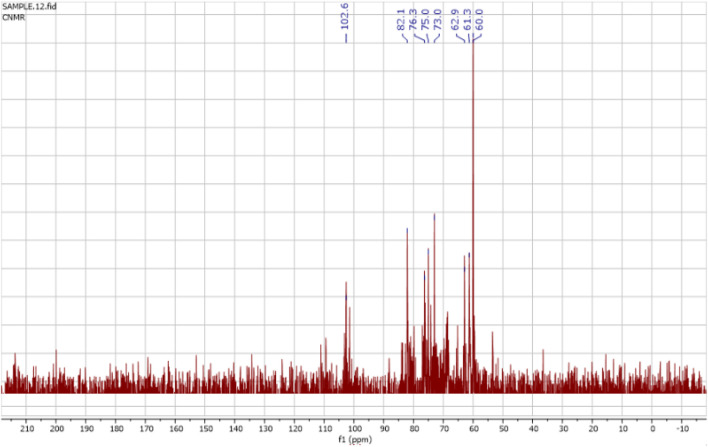
^13^C NMR spectrum of FAGAE.

The ^1^H NMR spectra of the FAGAE are shown in Figure 4 and are characteristic of protons from carbohydrate glycoside groups. The chemical shift of the anomeric protons is observed at δ 4.30 ppm–5.30 ppm (Hoang et al., 2024). The presence of double signals in the δ 4.38–4.43 and 4.90–5.30 ppm regions may correspond to the β- and α-anomeric proton configurations of arabinogalactans, respectively (Ghosh et al., 2015). The chemical shifts between δ 3.20 and 4.20 ppm were attributed to the glycoside ring protons (H2–H6). Resonances <3.0 ppm (Figure 4) were tentatively assigned to protons attached to nitrogen atoms in protein groups in a glucan–protein complex, which are typically observed in this region (Gonzaga et al., 2005). Furthermore, the ^13^C NMR spectra of FAGAE are shown in Figure 5, confirming the presence of carbons in glycoside groups. The chemical shifts of anomeric carbons were observed at δ 101 ppm–103 ppm. The signals at δ 60 ppm–80 ppm corresponded to C2–C6 of the glycoside rings (Karácsonyi et al., 1984; Kawagishi et al., 1990). Although the NMR of polysaccharides is complex due to spectral overlaps, the analysis described above can confirm the possible presence of arabinogalactan-type polysaccharides in FAGAE (Yao et al., 2021).

### 3.3 Open-field test

**FIGURE 6:**
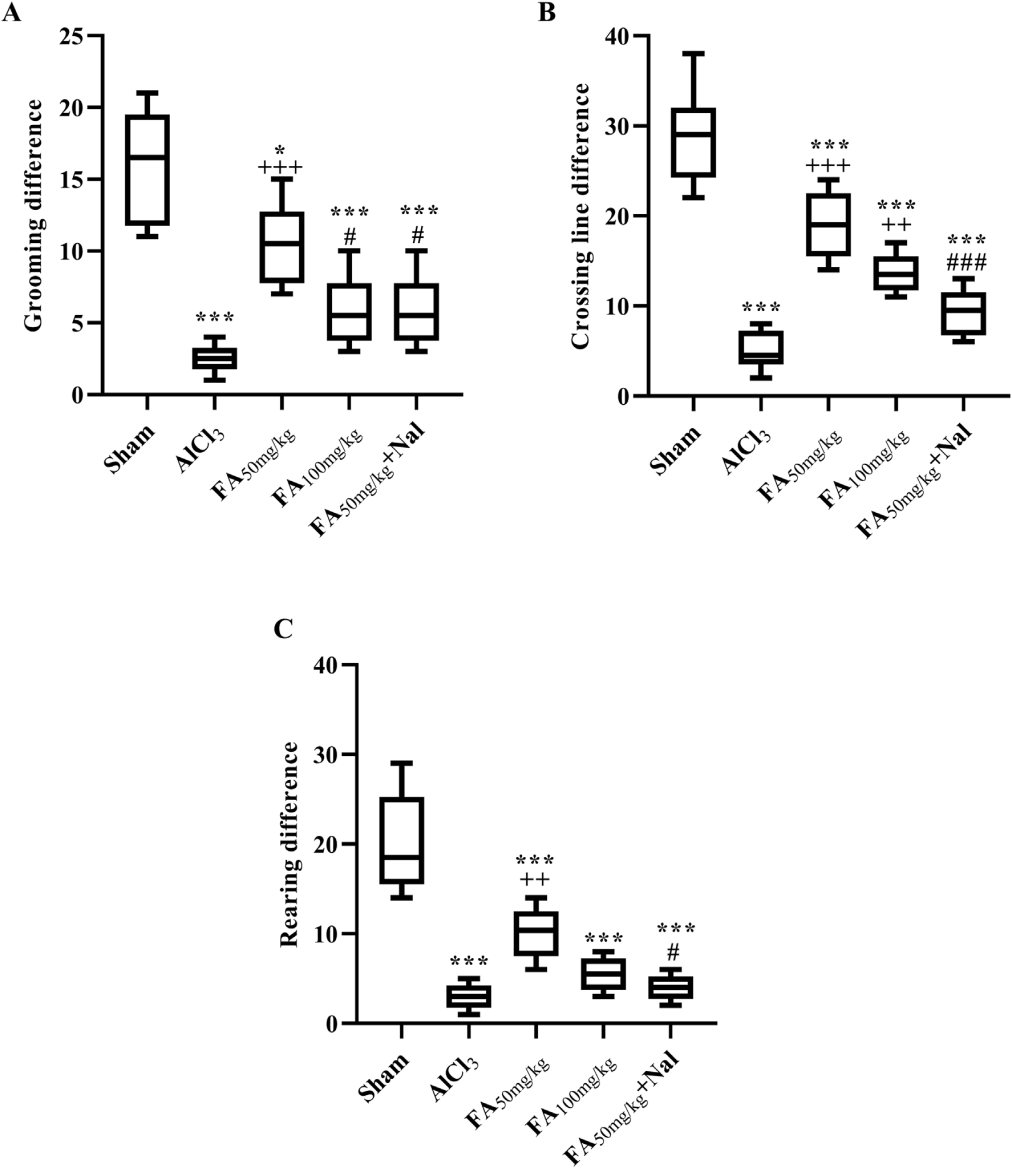
Effects of FAGAE and FAGAE + Nal on grooming differences **(A)**, horizontal exploration differences **(B)**, and rearing differences **(C)** in rats following AlCl_3_-induced AD. Data are presented as the mean ± SEM (*n* = 6). ^*^ *p* < 0.05 and ^***^ *p* < 0.001 vs. sham; ^++^ *p* < 0.01 and ^+++^ *p* < 0.001 vs. AlCl_3_; and ^#^ *p* < 0.05 and ^###^ *p* < 0.001 vs. FA_50_. Aluminum chloride (AlCl_3_), *Ferula ammoniacum* gum aqueous extract (FAGAE, shown as FA), naloxone (Nal).

The locomotor activity of rats with AlCl_3_-induced AD was recorded using the open-field test (Figure 6). In this test, the AlCl_3_ group exhibited a significant reduction in exploratory behaviors, both in peripheral areas (which indicate anxiety or fear responses) and central (horizontal) exploration, along with a significant decline in grooming and rearing activities. These findings suggested that AlCl_3_ led to behavioral deficits, reflecting impaired motivation and reduced exploration. In contrast, administration of FAGAE at 50 mg/kg significantly enhanced these behaviors compared with the AlCl_3_ group (*p* < 0.001). Specifically, this dose of FAGAE was more effective than a higher dose of 100 mg/kg in improving locomotor activities (Figure 6A: [F (4, 25) = 21.82, *p* < 0.001], Figure 6B: [F (4, 25) = 42.71, *p* < 0.001], and Figure 6C: [F (4, 25) = 30.83, *p* < 0.001]). Further investigations revealed that naloxone, an opioid receptor antagonist, partially reversed the positive effects of the 50 mg/kg dose of FAGAE on grooming, rearing, and crossing behaviors. This suggests that opioid signaling pathways are involved in the locomotor improvements associated with FAGAE (*p* < 0.05).

### 3.4 Passive avoidance test

**FIGURE 7:**
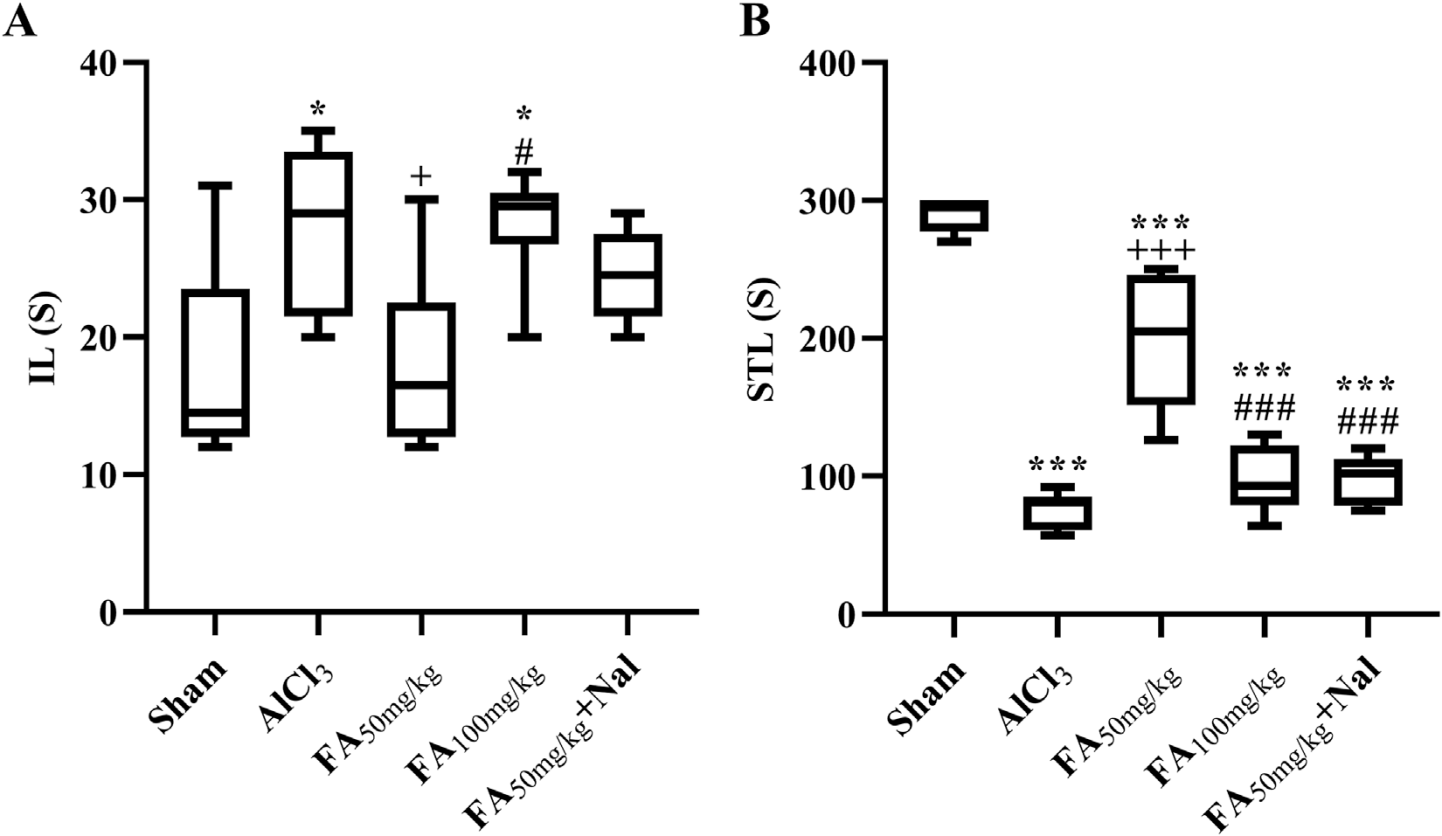
Effects of FAGAE and Nal + FAGAE on IL **(A)** and STL **(B)** in rats following AlCl_3_-induced AD. Data are presented as the mean ± SEM (*n* = 6). ^*^ *p* < 0.05 and ^***^ *p* < 0.001 vs. sham; ^+^ *p* < 0.05 and ^+++^ *p* < 0.001 vs. AlCl_3_; and ^#^ *p* < 0.05 and ^###^ *p* < 0.001 vs. FA_50_. Aluminum chloride (AlCl_3_), *Ferula ammoniacum* gum aqueous extract (FAGAE, shown as FA), naloxone (Nal).

In this study, exposure to AlCl_3_ initially increased IL (the time taken for the animal to enter the dark area) in the passive avoidance test, as shown in Figure 7A. The statistical results indicated that this effect was significant [F (4, 25) = 4.898, *p* = 0.0047], suggesting that AlCl_3_ may initially impair the animal’s ability to remember the avoidance behavior, possibly indicating some cognitive dysfunction or memory impairment. Conversely, Figure 7B shows that AlCl_3_ also decreased STL, indicating that the animals entered the area associated with the unpleasant stimulus more quickly [F (4, 25) = 68.47, *p* < 0.001]. This suggests that AlCl_3_ not only impaired memory but also increased impulsivity, leading to a faster response in entering the previously avoided area. The administration of FAGAE_50_ significantly decreased memory impairment induced by AlCl_3_. In addition, using an opioid antagonist reduced the effects of FAGAE_50_, which revealed the possible involvement of opioidergic pathways in the therapeutic effect of FAGAE (*p* < 0.001).

### 3.5 Elevated plus maze test

**FIGURE 8:**
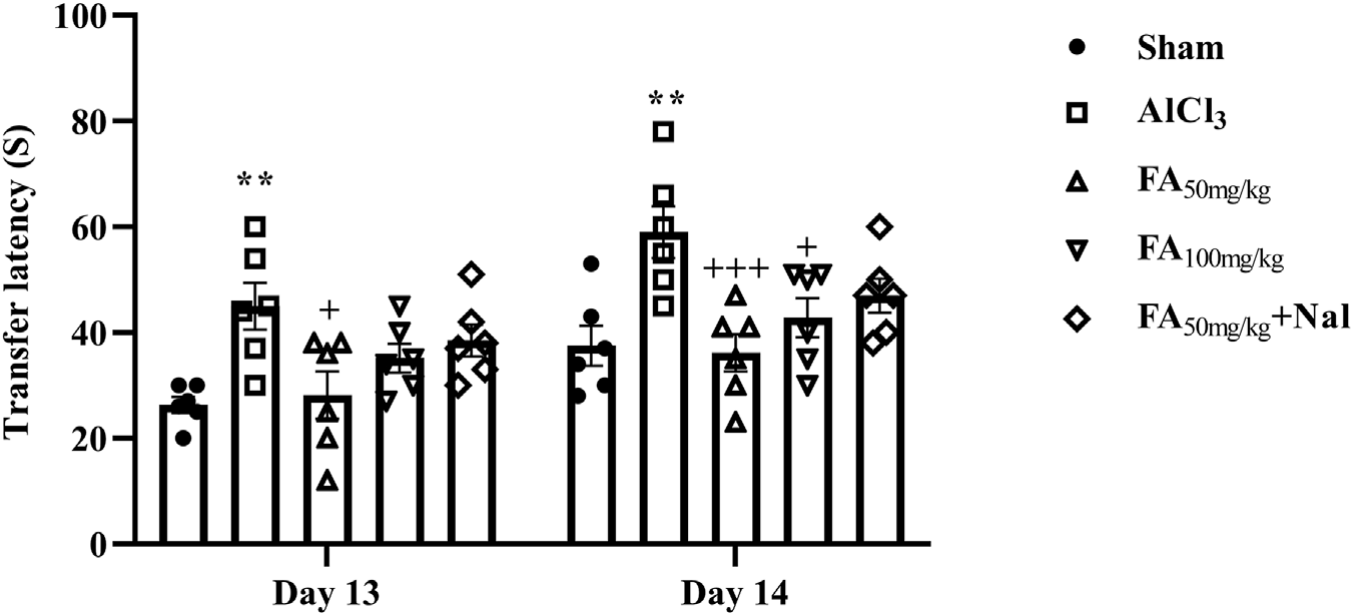
Effects of FAGAE and Nal + FAGAE on retention transfer latency in rats following AlCl_3_-induced AD. Data are presented as the mean ± SEM (*n* = 6). ^**^ *p* < 0.01 vs. sham; ^+^ *p* < 0.05 and ^+++^ *p* < 0.001 vs. AlCl_3_. Aluminum chloride (AlCl_3_), *Ferula ammoniacum* gum aqueous extract (FAGAE, shown as FA), naloxone (Nal).

This is a widely used experimental paradigm in rodent studies to assess anxiety-related behavior. The rats receiving AlCl_3_ exhibited a significant increase in retention transfer latency on days 13 and 14 of the test. This prolonged latency indicated heightened anxiety levels in these rats compared to the control group (sham), suggesting that AlCl_3_ exposure may induce anxiety-like behavior. On the other hand, FAGAE at a dose of 50 mg/kg decreased transfer latency compared with the AlCl_3_ group. This suggests that FAGAE enables the rats to spend more time in the open arms of the maze, indicating lower anxiety levels [Figure 8, F_interaction_ (4, 50) = 0.27, *p* = 0.89; F_day_ (1, 50) = 18.33, *p* < 0.001; F_group_ (4, 50) = 10.48, *p* < 0.001]. Additionally, naloxone administration partially reversed the anxiolytic effects of FAGAE. This suggests that the mechanism by which FAGAE reduces anxiety may involve the possible role of opioid receptors.

### 3.6 Body weight changes

In a two-way repeated-measures design to track the animal’s weight changes, the sham group exhibited a typical pattern of weight gain throughout the study (Table 1). As anticipated, AlCl_3_ resulted in weight loss during the first 2 weeks and markedly inhibited weight gain compared with that of the sham group (*p* < 0.01). It is worth noting that rats receiving FAGAE therapy showed greater weight gain and less weight loss than those receiving AlCl_3_ [F (4, 25) = 22.71, *p* < 0.001].

**TABLE 1:** Weight differences between groups on days 1 and 14.

Groups	Sham	AlCl_3_	FA_50 mg/kg_	FA_100 mg/kg_	FA_50 mg/kg_ + Nal
Weight difference	15/33 ± 2/51	−25 ± 4/63^a^	3/5 ± 3/50^b^	2/83 ± 2/15^a^ ^,^ ^b^	−13/16 ± 3/01^a^

The effects of FAGAE and Nal + FAGAE on weight differences in rats following AlCl_3_-induced AD. Data are presented as the means ± SEM (*n* = 6).

^a^ *p* < 0.001 vs. sham.

^b^ *p* < 0.001 vs. AlCl_3_.

Aluminum chloride, AlCl_3_; *Ferula ammoniacum* gum aqueous extract, FAGAE (shown as FA); naloxone, Nal.

### 3.7 Nitrite assay

**FIGURE 9:**
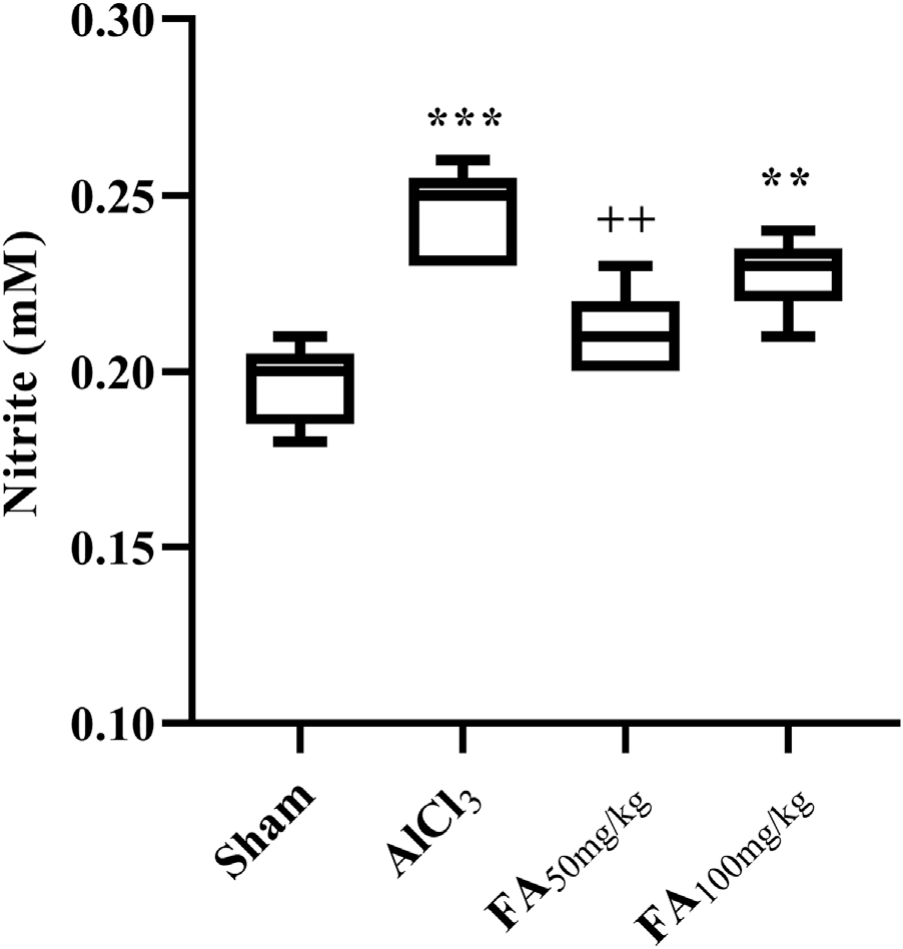
Effects of FAGAE on nitrite concentrations (mM) of rats following AlCl_3_-induced AD. Data are presented as the mean ± SEM (*n* = 5). ^***^ *p* < 0.001 vs. sham; ^++^ *p* < 0.01 vs. AlCl_3_. Aluminum chloride (AlCl_3_), *Ferula ammoniacum* gum aqueous extract (FAGAE, shown as FA).

Our results indicated a significant increase in the serum nitrite concentration in rats treated with AlCl_3_ [Figure 9; F (3, 16) = 15.11, *p* < 0.001]. Additionally, treatment with FAGAE decreased the nitrite concentration to the sham level (*p* < 0.01) (Figure 9).

### 3.8 MMP activity

**FIGURE 10:**
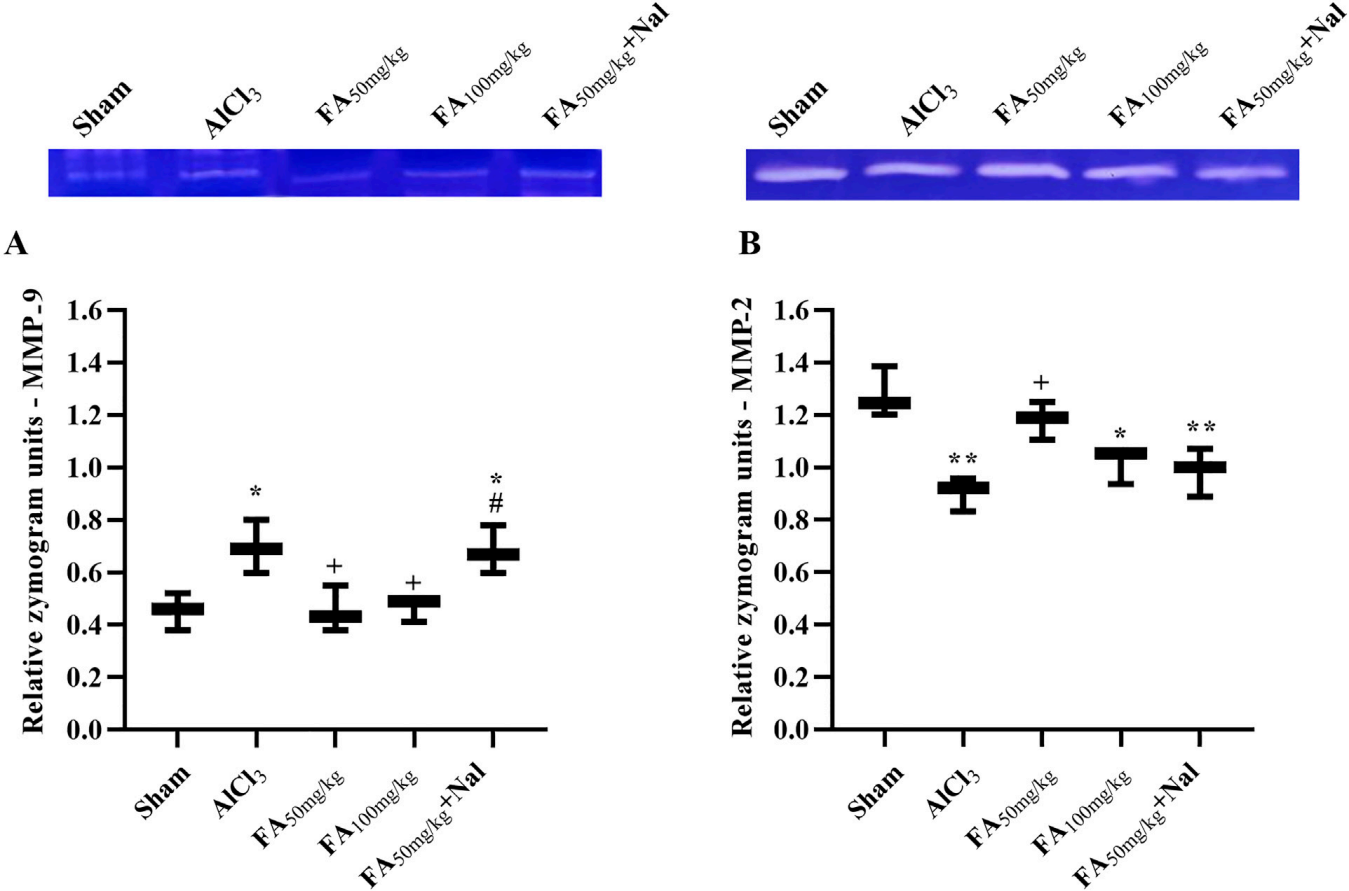
Effects of FAGAE and Nal + FAGAE on MMP levels in rats following AlCl_3_-induced AD. **(A)** MMP-9 and **(B)** MMP-2. Data are expressed as the mean ± SEM (*n* = 3). ^*^ *p* < 0.05 and ^**^ *p* < 0.01 vs. sham; ^+^ *p* < 0.05 vs. AlCl_3_; and ^#^ *p* < 0.05 vs. FA_50_. Aluminum chloride (AlCl_3_), *Ferula ammoniacum* gum aqueous extract (FAGAE, shown as FA), naloxone (Nal).

Compared with those in the sham group, zymography results indicated increased MMP-9 activity [F (4, 10) = 7.300, *p* < 0.05] and decreased MMP-2 activity [F (4, 10) = 10.99, *p* < 0.01] after AlCl_3_ treatment (Figure 10). Notably, both doses of FAGAE, particularly 50 mg/kg, showed protective effects. Furthermore, i.p. injection of Nal reduced the positive effects of FAGAE on MMP-2 and MMP-9 activity levels (*p* < 0.05).

### 3.9 Histopathological analysis

**FIGURE 11:**
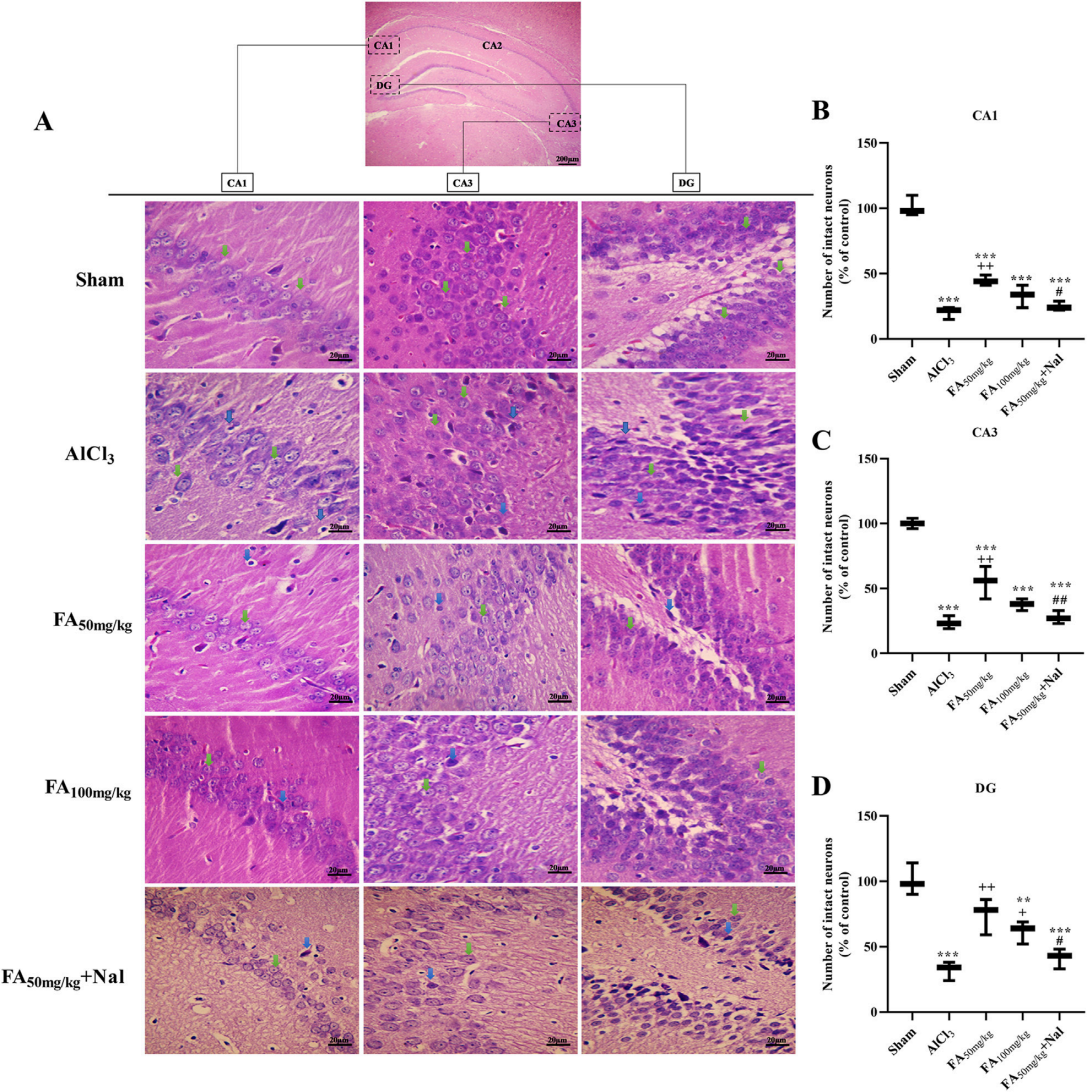
Effects of FAGAE and Nal + FAGAE treatments on histopathological changes in the hippocampus of rats following AlCl_3_-induced AD. **(A)** Representative H&E-stained neurons at ×400 magnification. The percentage of the number of control neurons was quantified in the **(B)** CA1, **(C)** CA3, and **(D)** DG regions. Data are presented as the mean ± SEM (*n* = 3). ^**^ *p* < 0.01 and ^***^ *p* < 0.001 vs. sham; ^+^ *p* < 0.05 and ^++^ *p* < 0.01 vs. AlCl_3_; and ^#^ *p* < 0.05 and ^##^ *p* < 0.01 vs. FA_50_. Aluminum chloride (AlCl_3_), *Ferula ammoniacum* gum aqueous extract (FAGAE, shown as FA), naloxone (Nal).

The results illustrated in Figure 11 indicate that following AlCl_3_ treatment, there was a notable reduction in the number of intact neurons in various hippocampal regions (*p* < 0.001). In contrast, the administration of FAGAE effectively preserved hippocampal neurons in the CA1 [F (4, 10) = 85.69, *p* < 0.001], CA2 [F (4, 10) = 59.36, *p* < 0.001], and DG [F (4, 10) = 21.11, *p* < 0.001] regions.

## 4 Discussion

**FIGURE 12:**
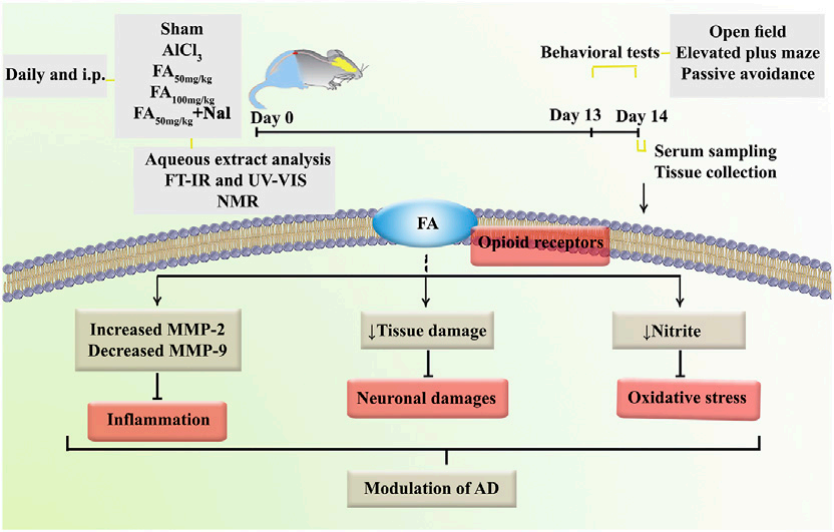
Overview of the research protocol to evaluate the mechanisms of FAGAE in combating AlCl_3_-induced AD in rats. Alzheimer’s disease (AD), aluminum chloride (AlCl_3_), *Ferula ammoniacum* gum aqueous extract (FAGAE, shown as FA), Fourier-transform infrared spectroscopy (FT-IR), intraperitoneal (i.p.), naloxone (Nal), nuclear magnetic resonance (NMR).

In the present study, we investigated the behavioral, biochemical, zymographic, and histopathological changes induced by AlCl_3_ in a rat model of AD. Additionally, we demonstrated alterations in the aforementioned variables following FAGAE treatment using open-field, elevated plus maze, and passive avoidance tests. We found that FAGAE ameliorated learning and memory impairments, serum nitrite concentration, MMP-2/MMP-9 activity, and hippocampus alterations in rats. Additionally, using an opioid antagonist partially reversed the anti-AD effect of FAGAE, which suggests the possible involvement of the opioidergic pathway (Figure 12).

Previously, evidence suggested that the anticonvulsant effects of FA act through benzodiazepine and opioid receptors, findings that align with our results. It has been previously reported that the moderate to advanced stages of AD result in a decline in motor abilities (Gould et al., 2009; Zidan et al., 2012; Serra-Añó et al., 2019; Van Ooteghem et al., 2019), which is consistent with our findings in a rat model of AlCl_3_-induced AD. The results of our study showed a relative improvement in the rats’ mobility parameters (e.g., grooming, rearing, and crossing) following FAGAE treatment.

Another behavioral test, the elevated plus maze, was used to evaluate learning and memory in rodents. In line with previous reports, results showed a decrease in AD rats’ performance on the elevated plus maze, highlighting the modulatory role of FAGAE. Earlier research demonstrated that AlCl_3_ causes a decline in STL and an increase in retention transfer latency in the elevated plus maze test, both of which were used to demonstrate the reduced performance in the passive avoidance test (Moreira-Silva et al., 2018; Salberg et al., 2019; Ai et al., 2020). The results of our study demonstrated that FAGAE delayed STL.

Weight changes, often manifesting as weight loss, have been reported in AD. Previous studies have demonstrated the relationship between increased mortality and greater severity of these changes in AD (White et al., 1998; Sergi et al., 2013). The results of our study showed that weight changes in the FAGAE-treated group were lower than those in the AlCl_3_ group, indicating an improvement in the relative status of the FAGAE-treated group. In almost all tests, a 50 mg/kg dose of FAGAE showed the most pronounced response, following a U-shaped dose–response curve, consistent with the concept of hormesis (Calabrese, 2008; Bahmani et al., 2025).

Considering the oxidative stress hypothesis of AD, elevated serum nitrite levels may activate downstream pathways of oxidative stress and inflammation. Oxidative stress arises from an imbalance between free radicals and antioxidants, leading to cellular damage. In addition, inflammatory responses typically lead to increased levels of reactive nitrogen species, such as nitrites. Consequently, monitoring nitrite levels can serve as a biomarker for evaluating the extent of oxidative stress and inflammation in various pathological conditions (Lundberg et al., 2009; Kapil et al., 2020). Given the near correlation between the oxidative and Aβ hypotheses, overproduction of Aβ leads to oxidative stress, which is a critical factor in AD pathology. This stress can promote the formation of peroxynitrite, a potent nitrating agent that can modify pathological proteins (Cheignon et al., 2018). The nitration of Aβ not only alters its aggregation properties but also increases its neurotoxicity by elevating intracellular calcium levels via interactions with N-methyl-D-aspartate (NMDA) receptors. This excitotoxicity can further exacerbate neuronal damage and increase nitrite levels as a byproduct of cellular stress responses (Tu et al., 2014; Guivernau et al., 2016). AlCl_3_ exposure increases oxidative stress and inflammation in neuronal cells. This is characterized by the generation of reactive oxygen species (ROS) and inflammatory mediators, which can damage cellular components, including lipids, proteins, and DNA (Liu et al., 2020). Studies have shown that AlCl_3_ can disrupt the balance of antioxidant enzymes, such as superoxide dismutase (SOD) and catalase (CAT), leading to increased lipid peroxidation and neuroinflammation (Hamdan et al., 2022). In previous reports, the same doses of FAGAE (50 and 100 mg/kg) showed anti-inflammatory (Pandpazir et al., 2018), antioxidant (Ahmadi et al., 2022), anti-seizure (Abizadeh et al., 2020), anti-nociceptive, and hypnotic effects (Jahani et al., 2020). In our study, FAGAE notably reduced the serum nitrite levels and MMP-2/MMP-9.

The Ferula genus contains diverse phytochemicals, including sesquiterpene chromandiones, coumarins, sesquiterpene derivatives, phenolic acids, flavonoids, and polysaccharides. Many compounds, such as *p*-coumaric acid, gallic acid, taxifoline, and catechin, exhibit neuroprotective and antioxidant effects (Appendino et al., 1991; Adhami et al., 2013; Mazaheritehrani et al., 2020). Polysaccharides from FAGAE have antioxidant and anti-inflammatory properties that contribute to AD improvement by modulating inflammation, oxidative stress, and related signaling pathways (Zhao et al., 2023). Similarly, the FAGAE FT-IR, NMR, and UV analysis showed the presence of polysaccharides with tentative arabinogalactan structures. Polysaccharides with the least side effects improved AD by targeting several mechanisms, including reducing inflammation, enhancing antioxidant activity, modulating anticholinergic signaling, and modulating other cell signaling pathways involved in AD progression (Bauer et al., 2021; Zhang et al., 2022; Tang et al., 2024). Recent studies indicate that traditional Chinese medicine-derived polysaccharides exert multifaceted effects on AD, primarily through interactions with gut microbiota and the gut–brain axis. Acting as prebiotics, these polysaccharides reshape microbial composition and promote the production of neuroactive metabolites, such as short-chain fatty acids, thereby modulating immune responses and neuroinflammation, leading to improved cognition and reduced biochemical markers of AD. They also exhibit multi-target therapeutic potential by reducing inflammation, enhancing antioxidant defenses, and inhibiting enzymes involved in amyloid processing and neural signaling (Wang et al., 2025).

From a mechanistic perspective and considering the roles of MMP-2 and MMP-9 in AD, our study showed that FAGAE regulates MMP activity in the serum. MMPs include Ca^2+^- and Zn^2+^-dependent endopeptidases that play essential roles in attenuating the degradation of extracellular matrix proteins, neurotransmitter receptors, growth factors, and cell surface components (Ethell and Ethell, 2007). In our study, FAGAE decreased inflammatory MMP-9 while increasing anti-inflammatory MMP-2 in the rat model of AD. Similar reports have also shown the role of MMP-9 in neuroinflammation, myelination, and blood–brain barrier penetration (Rosenberg, 2009). As previously reported, there is a near interconnection between MMP dysregulation and AD (Ethell and Ethell, 2007). Accordingly, previous works have focused on developing MMP-9 inhibitors as potential neuroprotective and anti-inflammatory compounds (Iranpanah et al., 2024). While MMP-2 plays a protective role and is suppressed during AD, MMP-9 is overexpressed and plays inflammatory roles (Fujimoto et al., 2008; Wang et al., 2014). On the other hand, Aβ reduced MMP-2 expression and promoted an inflammatory response in astrocytes during AD (Li et al., 2011; Wang et al., 2014). Consistent with these findings, zymography analysis showed elevated MMP-2 activity and reduced MMP-9 activity (Rosenberg, 2009; Gentile and Liuzzi, 2017). In line with these findings, gelatin zymography studies showed increased MMP-9 activity, but not MMP-2, in brains affected by AD and mild cognitive impairment (Lorenzl et al., 2007; Bruno et al., 2009). From another mechanistic perspective, previous reports have shown that dysregulation of opioid receptor signaling leads to the overexpression of γ-secretase and β-site amyloid precursor protein cleaving enzyme 1 (BACE1), both of which are critical for the production of toxic Aβ peptide and activation of the amyloidogenic pathway. This suggests that opioid receptors may impact amyloidogenic pathways and contribute to AD-associated neurodegenerative processes (Tanguturi and Streicher, 2023; Zarneshan et al., 2025). In the present study, using Nal, a non-selective opioid receptor antagonist, showed that FAGAE directly acts on opioid receptors to elicit neuroprotective responses.

From a histopathological perspective, the degeneration of hippocampal cells alters the structure of this tissue in AD, a process influenced by oxidative stress and Aβ deposition (Lichtenegger et al., 2018). AlCl_3_ causes neuronal degeneration in the hippocampus by downregulating the anti-apoptotic mediators and upregulating the pro-apoptotic factors (Niu et al., 2005). Chaudhary et al. (2014) found that Purkinje cells in the cerebellum were the most affected cell population, and their number decreased after AlCl_3_ treatment. In our study, FAGAE notably ameliorated neuronal degeneration in various hippocampal regions—CA1, CA3, and DG.

Since multiple mechanisms and hypotheses underlie AD pathogenesis, a critical limitation of *in vivo* models is that a single hypothesis is often prioritized as the primary goal. In contrast, other hypotheses remain as non-cores in the study. However, there are interconnections between the AD hypotheses that need to be studied further in future works. Future research should incorporate additional models or methodologies to explore other biochemical and pathological mechanisms of AD, including alterations in Aβ and tau protein levels, and the possible therapeutic role of FAGAE. On the other hand, given the presence of several major constituents in FAGAE, we hope our findings will encourage further research to provide a more comprehensive phytochemical analysis, identify its major bioactive constituents, elucidate the related pharmacological mechanisms in AD and other neurodegenerative disorders, and ultimately lead to well-controlled clinical trials. From another mechanistic perspective, cholinesterase inhibition is increasingly recognized as being linked to the core pathology of AD. Beyond symptomatic benefits, acetylcholinesterase accelerates Aβ aggregation, linking cholinergic deficits with plaque formation (Tripathi et al., 2024). This amyloid burden later triggers kinase activation and tau hyperphosphorylation, supporting the pathology of neurofibrillary tangle (Sharma et al., 2024). Recent studies have shown that plant-derived secondary metabolites (e.g., FAGAE compounds) act as multi-target neuroprotective agents by inhibiting cholinesterase activity, reducing Aβ toxicity, and attenuating tau-related signaling cascades (Siddiqui et al., 2024a; Siddiqui et al., 2024b). Altogether, these findings highlight the importance of integrating cholinesterase inhibition into the broader mechanistic aspects of AD pathophysiology. Regarding critical limitations, since naloxone is a non-selective antagonist of opioid receptors and antagonizes μ, δ, and κ receptors with different roles, further research is needed to critically unveil the specific type of opioid receptor involved in AD. Another limitation is that although AlCl_3_ specifically targets the cholinergic, oxidative, and inflammatory pathways, this AD model is not valid for tau and Aβ. In the AlCl_3_ model of AD, amyloid accumulation is inconsistent and weak, and it does not follow the human amyloidogenic pathway (Yenkoyan et al., 2025). In our report, the arabinogalactan-type polysaccharides in FAGAE showed promising anti-AD effects in an AlCl_3_ rat model. However, plant-derived active compounds appear to exhibit synergistic effects in an extract with minimal side effects. Future studies focusing on the formulation development/optimization/standardization, along with pharmacokinetic studies and brain bioavailability of the active compounds in FAGAE, are needed. Additionally, *in vivo* studies in aged animals and other animal models, followed by well-controlled clinical trials, are needed to confirm FAGAE’s acetylcholinesterase activity relative to conventional anti-AD drugs.

## 5 Conclusion

In conclusion, our study investigates the potential of FAGAE, a traditional medicinal plant, as a promising therapeutic option for AD. The results demonstrate that FAGAE modulates opioid receptors to alleviate behavioral impairments, reduce oxidative stress, suppress neuroinflammation, and ameliorate histopathological changes associated with AD and AlCl_3_ exposure.

## Data Availability

The raw data supporting the conclusions of this article will be made available by the authors, without undue reservation.
